# The epidemiology of hypopharynx and cervical esophagus cancer

**Published:** 2010-11-25

**Authors:** CR Popescu, SVG Bertesteanu, D Mirea, R Grigore, D Ionescu, B Popescu

**Affiliations:** * E.N.T. Department, Coltea Clinical Hospital, BucharestRomania; ** E.N.T. Department, University Emergency Clinical Hospital, BucharestRomania; *** E.N.T. Department, Coltea Clinical HospitalRomania

**Keywords:** Hypopharynx, cervical esophagus, cancer, epidemiology, risk factors, descriptive studies, analytical studies, experimental studies

## Abstract

At the beginning of the 21st century hypopharynx and cervical esophagus cancer represents a major issue for all countries of the world. The epidemiology of the hypopharynx and cervical esophagus cancer deals with the spread of the disease in human population in regards to sex, age, profession, time and space, as well as risk factors that contribute to these phenomena.

The main goal is to investigate the causes and the factors involved in the development of the tumors at the pharyngo–esophageal junction, knowledge that contributes to latest therapeutic assessment through interdisciplinary collaboration (E.N.T. surgeon, general surgeon, radiation oncologist, chemotherapist, nutritionist). The epidemiology of the hypopharynx and cervical esophagus cancer includes three major areas of interest: descriptive (the study of the spread in mass population), analytical (the study of causal risk factors on the disease) and experimental (that verifies by experiments on animals the prior identified hypothesis).

## Introduction

At the beginning of the 21st century hypopharynx and cervical esophagus cancer represents a major issue for all countries of the world. The epidemiology of the hypopharynx and cervical esophagus cancer deals with the spread of the disease in human population in regards to sex, age, profession, time and space, as well as risk factors that contribute to these phenomena.

The main goal is to investigate the causes and the factors involved in the development of the tumors at the pharyngo–esophageal junction, knowledge that contributes to latest therapeutic assessment through interdisciplinary collaboration (E.N.T. surgeon, general surgeon, radiation oncologist, chemotherapist, nutritionist). The epidemiology of the hypopharynx and cervical esophagus cancer includes three major areas of interest: descriptive (the study of the spread in mass population), analytical (the study of causal risk factors on the disease) and experimental (that verifies by experiments on animals the prior identified hypothesis)[10].

## Descriptive epidemiology

Hypopharynx and cervical esophagus cancer are usually being studied together because of the anatomic relation between the two, thus making the tumoral lession invade both regions at the moment of diagnostic, that is usually too late. Because of this the means of therapy (total cervical esophagectomy with total laringectomy, chemotherapy, radiation therapy) and the post–therapy oncological follow–up are similar. As much as 95% hypopharynx and cervical esophageal cancer are squamous carcinoma. Adenocarcinoma may also be encountered. In present times there are over 200 polpulation registries including the esophageal and hypopharynx cancer registry that evaluates the incidence and allows following up the evolution of mortality in time, in relation with specific treatment.

There are differences in geographical distribution of the hyphopharynx cancer, the analysis of the data being complex because of the lack of a subclassification of different anatomic regions (piriform sinus, posterior wall, post–cricoid region). For example post–cricoid cancer differ from other hypopharynx cancers in terms of geographic spread, male/female distribution, risk factors. The average annual incidence is 1:100,000 inhabitants. Increased incidence in males of over 2.5:100,000 is seen in India, Brazil, Central and Western Europe and decreased incidence under 0.5:100,000 in Eastern Asia, Africa and Northern Europe. France and India record thegreatest incidence in male population 8 to 15 cases in 100,000 population. The incidence in women is as high as 0.2:100,000 in the majority of the countries, except for India (1:100,000). 

The American Cancer Society (Jemal et al. 2006) describes 30,990 new cases and 7430 deaths in people with pharynx and oral cavity cancer, without making the difference between tumors of the rhino–, oro–, and hypopharynx. The same analysis shows 14,550 new cases and 13,770 deaths induced by esophageal cancer [18]. In the U.S.A. hypopharynx cancer is not a common disease representing, along with the cervical esophageal cancer, as much as 10% of the tumors of the superior aerodigestive tract and less than 1% of the year–long diagnosed cancers [24]. There are two database in U.S.A. that reveal the incidence of the hypopharynx cancer without including the cervical esophagus cancer. Analyzing data from the National Cancer Database Hoffman et al. estimate that hypopharynx neoplasia represents 4.3% of the head and neck cancer in one study made between 1985 and 1994 [13]. 

Canto and Devesa analyzed data from nine Epidemiology Registries, survival and death rates (SEER) between 1972–1988 and concluded that there is a sex/rase related incidence concerning hypopharingeal cancer, the study referring to hypopharynx and piriform sinus (Table 1)[5].

**Table 1:** Appearance rate on 100,000 persons/year of hypopharynx cancer regarding sex and rase (after Canto and Devesa 2002).

Sex-Rase/ Location	Hypopharynx	Piriform sinus
White male	0.4	0.9
Black male	0.8	2,3
White women	0.2	0.2
Black women	0.2	0.5

Hypopharynx cancer usually occurs in the second half of life, between 50–79 years, more frequent in males. There have been described pharyngeal cancers in children. An increased incidence of post–cricoid cancer has been encountered in women with Plummer–Vinson syndrome from anglo–saxon countries. Some authors (Wahlberg 1998) by analyzing statistics from 1960–1989 period in Sweden noticed a rate of 1.22:100,000 in males and 0.45:100,000 in women with hypopharynx cancer. These average rates have changed to an increasing ratio male/female because of the sideropenic anemia and Patterson–Kelly syndrome therapy part of some national prophylactic health programs. In some countries, in the last two decades, we find a decreased male/female ratio due to an increased smoking rate in women, knowing that smoking is a major risl factor for pharyngo–esophageal neoplasia. Concurrent with this pathology effemination we find a downward readjustment of the age of appearance of the pharyngo–esophageal neoplasia because of the early introduction of smoking in the individual habits [20]. Cervical esophageal cancer represents 5,3% of all esophageal cancers, usually caudal extended from hypopharingeal segment. Male incidence is increasing from 1975 with a rate of 1,7%/year with a constant modification of proportion between different histipathological types and location of esophageal cancer. In the regions between Iran, Central Asia, Mongolia and Northern China the incidence is 10–100 times greater than in the countries with low risk.

Hypopharynx and esophageal cancers are present in countries with low social and economical standards and with low level of education.

In Romania, in the last decades, the global frequency of cancer has risen, being second place after cardio–vascular diseases. According to statistics from 1996 regarding males the incidence of pharyngeal cancer was third (11.52:100,000) after pulmonary and stomach cancers. In Cluj county in 2000 esophageal cancer was reported with an incidence of 1.2:100,000 in men and 0.2:100,000 in women. Global mortality by malign tumors, taking 1958–1998 period into study, has risen by 33.9% in men and for pharyngeal cancer with 23% despite modern means of treatment. there has been an increase of mortality in city areas in comparison with rural areas [21]. 

This increased mortality is also associated with late diagnostic of hypopharynx and cervical esophagus cancer, 77.3% of the patience being diagnosed with stage 3 or stage 4 at admittance. Because of this the 5–year survival rate is extremely low approximately 17–30%, in comparison with other primary sites of origin of head and neck region.

The general tendency is that of rising incidence of the hypopharynx and cervical esophageal cancer in both women and men, by increasing tobacco and alcohol consumption. Thus the first epidemiological preventive measure should be prohibition or decreased tobacco and alcohol consumption.

## Analytical epidemiology

The apparition of hypopharynx and cervical esophageal cancer  is frequently associated with a series of risk factors. As in most head and neck neoplasia excessive consumption tobacco, alcohol, in association with genetic, alimentary and occupational factors, as well as preexistent pathological lesions are incriminated in the appearance of malignant hypopharingo–esophageal tumors. Knowing this is of most importance for the prophylactic and therapeutic approach, the elimination of one or more risk factors can result in decreased incidence of this poor prognostic disease.

**Tobacco consumption** represents the most frequent cause of head and neck tumors. Neoplastic histopathological modifications occur because of the direct contact of tobacco, carcinogenic substances from tobacco and smoke inhaled in the upper respiratory airways with the pharyngo–esophageal mucosa. A number of cohort and case–control studies reveal the close connection between increased incidence and mortality by hyopharynx and cervical esophageal cancer in comparison with non–smoking individuals. Increasing apparition risk of neoplasia is in close connection with the quality of tobacco, way, duration of smoking and association with other predisposing factors (alcohol, asbestosis, occupation). Doll et al. (1994) support the idea that the mortality rate by esophageal cancer is 15 times higher in great smokers than non–smoker individuals [9]. 

Early debut of smoking, consumption of a great number of cigarettes per day and a deep inhalation lead to an increased risk of pharyngo–laryngeal neoplasia. An average consumption of about 20 grams/day with a history of 300 kg prediagnostic consumption is frequently encountered in patients with hypopharynx cancer. Likewise the use of black tobacco is far more dangerous than yellow tobacco use. Studies from India show the association between hypopharyngo–esophageal squamous cell carcinoma with chewing or snuffing tobacco or other tobacco products. There is a multitude of evidence on the greater risk of developing pharyngeal and esophageal cancer by the regular smoking individuals than the occasional smokers. According to the U.S.D.H.H.S. the risk of apparition of cancer decreases after 3–4 years for occasional smokers and after 10 years for regular smokers.

Alcohol consumption is in close causal relation with oral, pharyngeal, laryngeal, esophageal tumors. Smoking and alcohol consumption rise the risk level up to as much as 100 times greater than regular non–smoking no–alcohol consuming individuals for developing neoplasia in superior aero–digestive tract. Alcohol alone can increase the risk of developing tumors in aero–digestive tract in non–smoking patients. A patient with an average consumption of about 150 g/day and a total of 1.5 tons of alcohol has an increased risk for developing tumors in the hypopharynx and esophageal area. Genetic mutation in alcohol–dehydrogenase 1B (ADH1B) and aldehyd dehydrogenase–2 (ALDH–2), involved in the metabolism of alcohol can result in the development of hypopharynx neoplasia. Epidemiologists attribute as much as 2–4% of cancer deaths to alcohol. The consumption of strong alcohol beverages in a medium to high quantity for a long period of time rises the proportion of hypopharynx and cervical esophageal cancers. According to IARC 1998 the type of consumed alcohol in a certain region in the world can influence the incidence of esophageal cancer: calvados (northern France), house rum (Puerto Rico), cachaca (Brasil) [1,3,16]. 

**Occupational factors** have been long studied to clarify the level of involvement in the development of neoplasia independent to alcohol and tobacco consumption. The issue of industrial exposure is hard to be evaluated because of a high incidence if pharyngo–esophageal neoplasia in unqualified workers in agriculture and industry. These socio–economical categories are frequently associated with tobacco and alcohol consumption, thus is hard to evaluate the degree of involvement of the occupational factors. None the less the link between massive exposure to toxics, different jobs and hypopharynx and cervical esophageal cancers has been demonstrated by a series of studies. In medical literature there is quotation of a great number of cases of neoplasia attributed to workers in rubber industry, ethiological agents of asbestosis, sulfuric acid [14,15,19]. According to Boffett et al. (2003) in one study made between 1980–1983 in France, Italy, Spain and Switzerland in 1010 cases of pharyngo–laryngeal carcinoma and 2176 patients that have been investigated we find that there is a correlation between the level of risk of cancer development and occupational factors. Thus the risk is greater for iron workers (OR 3.7, 95% CI 1.2–11.9, 5 cases), masons (OR 6.9, 95% CI 1.2–41.0, 4 cases), unqualified workers (OR 1.6, 95% CI 1.1–2.2, 61 cases), railway workers (OR 3.0, 95% CI 1.1–8.2, 7 cases), metal workers (OR 2.4, 95% CI 1.1–5.3, 11 cases), cement industry workers (OR 4.1, 95% CI 0.9–19.4, 3 cases), rock diggers (OR 3.7, 95% CI 0.8–16.1, 3 cases). The study also reveals that administrators, managers, salesmen, fire workers have a low risk of developing pharyngo–esophageal neoplasia. A very important fact is the time of exposure to different toxic industrial substances this being in direct correlation to the development to pharyngo–esophageal neoplasia. The occupational factors is responsible for 2–4% of all cancer related deaths. The development of pharyngo–esophageal neoplasia is in relation to occupational factors, toxic exposure (asbestos fibers) and other risk factors (tobacco, alcohol). 

**Exposure to asbestos fibers** determines an increase in the incidence of pulmonary cancers, mesothelioma, and digestive cancers. The association between hypopharynx cancer and asbestosis has been described in 16 cohort studies and 6 case–control studies. The time of exposure to asbestos fibers as well as tobacco and alcohol association has been taken into consideration when establishing the results of the studies (Table 2).

**Table 2:** Association between pharynx cancer and exposure to asbestos fibers and tobacco (RR – relative risk, CI – trust interval)(modified after Committee on Asbestos: Selected Health Effects 2006).

Study	Smoking history (packs/year)		RR with 95% CI for asbestos exposure
		None / Small	Intermediate /High
Marchand et al. (2000) (adjusted on age and alcohol consumption)	< 30	1.0	1.2 (0.6 – 2.3)
	30 +	4.0 (2.2 – 7.2)	6.2 (3.4 – 11.4)

The combination asbestos–tobacco has been determined as risk factor for lung cancer, but for hypopharynx cancers there is only one case–control study that suggests asbestos exposure as a cofactor along with tobacco. This fact is due to differences between oro– and hypopharynx mucosa and tracheal, bronchi epithelium. Modern data is suggestive but not sufficient to establish a casual relation between asbestos exposure and hypopharynx cancer. Cohort studies tend to rise controversies on esophageal cancer if Berry (2000) describes great increase in the risk for cancer development associated to any exposure to toxic agents, Meurman (1994) finds a dose–response relation. Therefore a number of studies indicate the association between asbestos exposure and cervical esophagus cancer but the evidence are inadequate for establishing a cause–effect relation [6]. 

The variable geographic distribution of cancer suggests an influence from nutritional factors in the apparition of neoplastic disease. There have been formulated several hypothesis in regards to the alimentation factors in the attempt to explain the differences in incidence between different countries. The hypopharynx and cervical esophageal cancer tends to respect the same geographic distribution. We previously demonstrated how certain local beverages consumption (calvados, cachaca, dark rum, mate) is associated with a high incidence of hypopharynx cancer. Toxic agent produced by Fusarium moniliforme (B1, B2, fumonisins, C–fusarin), fungus present in cereals, appear to play a key role in the pathogenesis in human cervical esophageal cancer. There are no data yet on the percentage of fusarium present in aliments. Along with this nitroseamines are also involved in the increase of esophageal cancer. High consumption of salted aliments (pickles, brine) or moldy aliments, nitroseamine reserves, in high risk area in China shows their involvement in the etiopathogenesis of squamous–cell esophageal cancer [30]. Diet habits can also have a protective role. As in the colon cancer matter a high vegetables–fruits fiber diet seems to have a preventive effect on pharyngo–laryngeal neoplasia. Specific studies on the protective role of carotenoids and vitamin C show inadequate evidence to sustain this conclusion. A series of controversies have arisen in regards to selenium level in aliments and the incidence of esophageal cancer. In one study conducted in South Africa by Jaskiewicz et al. 1998 it is shown that while black population living in the esophageal cancer risk areas have a low blood level of selenium (58–71 ng/ml) the population living in low risk areas have increased blood selenium levels (114–177 ng/ml)[17]. Profilactic iron administration in bakery products in anglo–saxon countries leaded to a decrease in sideropenic anemia associated with Plummer–Vinson syndrome and there for a decrease in post–cricoid cancer especially present in female population. Low calories, low protein, low fat diets that are common in poor economy areas increase the risk of esophageal cancer. Excessive fat consumption can lead to obesity this being a promoting agent for the appearance of adenocarcinoma of the esophagus.

Genetic predisposition is taken into consideration in patients with hypopharynx and cervical esophageal cancer that are not exposed to regular risk factors (tobacco, alcohol). We can find genetic abnormalities in enzymes that determine:

- changes in tobacco metabolism (transforming liposoluble derived carcinogens into hydrosoluble ones with a good intracellular penetration) and other carcinogens
- chromosomal mutation sensibility with the alteration of the repairing process of the DNA.

Genetic polymorphism of enzymes involved in alcohol metabolism (ADH1B, ALDH2) is incriminated in carcinogenic process in hypopharynx. Suppressive  gene p53 mutation has been detected in adenocarcinoma, epidermoid carcinoma and esophageal displasia [26]. According to Twu (2006), in one study made in Taiwan on 53 patients with hypopharingeal cancer and 53 control patients, the polymorphism of p53 72 pro homozygouse codone is associated with high incidence of hypopharingeal cancer. Tumor genesis related identification and metastasis potential of hypopharingeal cancers is of great importance in patients staging in prognostic groups and selection for aggressive treatment. Cromer (2004) reports the presents of 119 genes with expression modification in early hypopharingeal tumors in comparison with normal tissue [8]. Multiple genetic modifications have been determined in squamous cell carcinoma of the esophagus [12](Table 3).

**Table 3:** Genetic modifications in squamous esophageal carcinoma (modified after Hamilton SR, Aaltonen LA, Pathology and Genetics of Tumors of the Digestive System, OMS, IACRS 2000, 11–19).

Genes	Location	Tumor abnormality	Function
DLC1	8p21.3	Transcription stopping	Growth factor
FEZ1	8p22	Transcription stopping	Transcription factor
c–myc	8q24.1	Amplification	Transcription factor
p16, p15, ARF/CDN2	9p22	Homozygous inhibition	CDK inhibitor (cellular cycle control)
Cyclin D1	11q13	Amplification	Cellular cycle control
RB	13q14	LOH, absence of expression	Cellular cycle control
TP53	17p13	Punctiform mutation, LOH	Apoptosis, genetic stability, G1 stopping
EGFR	17p13	Amplification, over on	Transduction signal
TOC	17q25	LOH	Transduction signal

Genetic mutations have been observed in patients with multiple primary cancers in the ahead and neck area as well as in patients with family history for cancers in the ENT areas. Approximately 35% of the patients with family history of head and heck neoplasia have a second cancer in the superior aero–digestive tract. Family aggregation in hypopharynx and cervical esophagus cancer has been demonstrated in one study conducted by Morita et al. 1998 at Kyushu National Cancer Institute on 167 patients. 18 patients had superior aero–digestive cancer, 37 patients had family history for head and neck cancer and 112 patient that had heredo–colateral antecedents of cancer. In that particular study we can observe an increased risk of developing hypopharynx ancd cervical esophagus cancer especially in patients with family history of head and neck neoplasia. The age of appearance of hypopharingo–esophageal cancer is a lot lower for patients with family history of head and neck neoplasia [22]. 

The presence of associated or precursor pathology of hypopharynx and cervical esophagus cancer has been shown in high risk area for this kind of localization. The connection between Plummer–Vinson (Paterson–Kelly) syndrome and post–cricoid cancer in anglo–saxon countries is well known. Upper esophagus opening cancer can appear from a degenerative pharyngo–esophageal diverticulum, 2,5% anatomopathological neoplasia modification on corrosive esophagitis according to Piquet 1958, invasion from a nearby cancer (regional extension of a hypopharynx or thoracic esophagus tumors, or distant metastasis) [25]. The presence of cancer in the upper aero–digestive tract increases the probability of a hypopharingeal and cervical esophagus cancer. Tylosis, hand and foot hyperkeratosis have been associated with spinocelular cancer in the upper esophagus. Gastroesophageal reflux, achalasia, obesity, Barett esophagus are incriminated in the increased risk of appearance of esophageal carcinoma.

The appearance of the hypopharynx and cervical esophagus cancer in the context of HPV infection has not yet been demonstrated, studies being inconsistent. None the less HPV DNA has been found in 20–66% of spinocellular esophageal carcinoma in high risk areas in China. The main strains involved are HPV 16 and 18. The differences of HPV involvement in the spinocellular esophageal cancer genesis are explained by the different detection techniques applied to various risk population groups. Recent studies show the protective role of gastric Helicobacter pylori on the appearance of esophageal adenocarcinoma. Ebstein–Baar virus is involved in hypopharynx lymphoepitelioma apparition [11].

## Experimental epidemiology

In time there has been conducted a number of experiments on animals in order to demonstrate the involvement of risk factors in the etiology of hypopharynx and cervical esophagus cancer. Pure alcohol is not a carcinogen by itself on test animals. Carcinogenic effect is due to tissue destruction (liver cirrhosis) or due to facilitating carcinogenic substances penetration by tissue exposure (oral, pharynx, esophageal cancers). Birt (1986) observes the association between lack of selenium and cancer in animals. It has been demonstrated that chronic tobacco smoke exposure leads to neoplastic changes whereas asbestos fibers chronic exposure does not lead to the appearance of pharyngo–esophageal tumors in animals [2].

Present data show the need for further studies on risk factors implication in the development of hypopharynx and cervical esophagus cancer and on mortality and morbidity influence on age and sex groups. Further scientific data are to be disclosed in a series of articles.
